# H_2_ drives metabolic rearrangements in gas-fermenting *Clostridium autoethanogenum*

**DOI:** 10.1186/s13068-018-1052-9

**Published:** 2018-03-01

**Authors:** Kaspar Valgepea, Renato de Souza Pinto Lemgruber, Tanus Abdalla, Steve Binos, Nobuaki Takemori, Ayako Takemori, Yuki Tanaka, Ryan Tappel, Michael Köpke, Séan Dennis Simpson, Lars Keld Nielsen, Esteban Marcellin

**Affiliations:** 1 Australian Institute for Bioengineering and Nanotechnology (AIBN), The University of Queensland, St. Lucia, Australia; 2 LanzaTech Inc., Skokie, USA; 3 Thermo Fisher Scientific, Bio21 Institute, The University of Melbourne, Parkville, Australia; 4 Proteo-Science Center, Ehime University, Ehime, Japan; 5 Advanced Research Support Center, Ehime University, Ehime, Japan; 6 Queensland Node of Metabolomics Australia, The University of Queensland, St. Lucia, Australia

**Keywords:** Acetogen, *Clostridium autoethanogenum*, Gas fermentation, H_2_ metabolism, Genome-scale modelling, Quantitative proteomics, Metabolomics

## Abstract

**Background:**

The global demand for affordable carbon has never been stronger, and there is an imperative in many industrial processes to use waste streams to make products. Gas-fermenting acetogens offer a potential solution and several commercial gas fermentation plants are currently under construction. As energy limits acetogen metabolism, supply of H_2_ should diminish substrate loss to CO_2_ and facilitate production of reduced and energy-intensive products. However, the effects of H_2_ supply on CO-grown acetogens have yet to be experimentally quantified under controlled growth conditions.

**Results:**

Here, we quantify the effects of H_2_ supplementation by comparing growth on CO, syngas, and a high-H_2_ CO gas mix using chemostat cultures of *Clostridium autoethanogenum*. Cultures were characterised at the molecular level using metabolomics, proteomics, gas analysis, and a genome-scale metabolic model. CO-limited chemostats operated at two steady-state biomass concentrations facilitated co-utilisation of CO and H_2_. We show that H_2_ supply strongly impacts carbon distribution with a fourfold reduction in substrate loss as CO_2_ (61% vs. 17%) and a proportional increase of flux to ethanol (15% vs. 61%). Notably, H_2_ supplementation lowers the molar acetate/ethanol ratio by fivefold. At the molecular level, quantitative proteome analysis showed no obvious changes leading to these metabolic rearrangements suggesting the involvement of post-translational regulation. Metabolic modelling showed that H_2_ availability provided reducing power via H_2_ oxidation and saved redox as cells reduced all the CO_2_ to formate directly using H_2_ in the Wood–Ljungdahl pathway. Modelling further indicated that the methylene-THF reductase reaction was ferredoxin reducing under all conditions. In combination with proteomics, modelling also showed that ethanol was synthesised through the acetaldehyde:ferredoxin oxidoreductase (AOR) activity.

**Conclusions:**

Our quantitative molecular analysis revealed that H_2_ drives rearrangements at several layers of metabolism and provides novel links between carbon, energy, and redox metabolism advancing our understanding of energy conservation in acetogens. We conclude that H_2_ supply can substantially increase the efficiency of gas fermentation and thus the feed gas composition can be considered an important factor in developing gas fermentation-based bioprocesses.

**Electronic supplementary material:**

The online version of this article (10.1186/s13068-018-1052-9) contains supplementary material, which is available to authorized users.

## Background

In the face of a warming planet and more inclement climate, the world faces an increasing need to drastically reduce its carbon usage. However, many fuels and chemicals are carbon-based and, therefore, not positioned to be phased out in a world less dependent on fossil fuels. Thus, fuels and chemicals must come from renewable and sustainable feedstocks. Gas fermentation shows potential in being part of this solution [1] and it has received considerable interest for converting inexpensive and abundant gaseous waste feedstocks (e.g., syngas [CO, H_2_, and CO_2_] from gasified biomass, industrial waste gases) into valuable fuels and chemicals [2–5], as opposed to the currently dominating fossil-based industries. Importantly, gas fermentation utilises non-food-based feedstocks and offers high product versatility [4].

Acetogens are the preferred organisms for gas fermentation, since they can use gas as their sole carbon and energy source [6]. They employ the Wood–Ljungdahl pathway (WLP) to convert CO or CO_2_ (with H_2_) into acetyl-CoA [6, 7], making the pathway the most, and only, energy-efficient linear pathway for the synthesis of acetyl-CoA from CO_2_ [8–10]. All acetogens can natively produce acetate, while the autotrophic spectrum of other products (e.g., ethanol, butyrate, and 2,3-butanediol) varies between species [4]. Notably, during autotrophic growth on CO, the more energetically and thermodynamically favourable carbon source [10, 11], a fraction of carbon is dissipated as CO_2_ to generate extra reducing equivalents by CO oxidation. This loss of carbon can, however, be diminished by generating redox from H_2_ oxidation by cellular hydrogenases. In addition, H_2_ supplementation should enable elevated production of reduced and energy-intensive products. For the latter reasons, H_2_-rich gas streams (e.g., syngas) sourced from biomass, industrial, or municipal waste [12, 13] are attractive feedstocks.

The benefit of H_2_ supplementation has been analysed on the theoretical stoichiometric conversion of CO into ethanol [5, 11, 14]. While 2/3 s of CO is lost as CO_2_ during ethanol formation, when H_2_ is not present, less carbon needs to be dissipated as CO_2_ with increasing H_2_ availability. Theoretically, no carbon should be lost as CO_2_ above an H_2_/CO uptake ratio of two [14]. However, this would come at an expensive thermodynamic cost as the Gibbs free energy change decreases with higher H_2_ utilisation [14].

Despite the relevance of H_2_-rich gas streams, to the best of our knowledge, the effect of H_2_ supplementation on CO-grown acetogens has yet to be experimentally quantified under controlled conditions, due to experimental limitations. The probable reason is that co-utilisation of CO and H_2_ is not observed in most batch cultures until CO is almost completely consumed [11, 15–17]. This is because acetogens’ Fe-based hydrogenases are strongly inhibited by even relatively low concentrations of CO [18–20]. For example, no co-utilisation of CO and H_2_ was detected when *Butyribacterium methylotrophicum* was grown on CO, syngas, or CO + H_2_ [17]. Although co-utilisation of CO and H_2_ was measured during *Alkalibaculum bacchi* [21] and *Acetobacterium*-like isolate [22] batch growth on various CO-containing gas mixes, uncontrolled growth conditions resulted in changing pH and specific growth rate (*µ*), making data interpretation challenging. Finally, no gas uptake data are available for comparison between *Clostridium autoethanogenum* growth on CO and syngas [23]. There are indications though from mixotrophic (fructose + H_2_) and co-culture experiments using acetogens that supply of H_2_ lowers the carbon flux to CO_2_ and acetate [24, 25] while increasing flux to reduced products [24].

Continuous cultures, however, show simultaneous utilisation of CO and H_2_ [26–28] and enable studying cells in controlled physico-chemical conditions, making continuous cultivation a suitable culturing method for investigating the effects of H_2_. In addition, continuous cultures yield steady-state data which are preferred for quantitative description and in silico reconstruction of metabolism [29, 30]. Therefore, the aim of our work is to perform the first quantitative molecular analysis of the effects of H_2_ supplementation on CO-grown acetogens using continuous cultures.

Here, we investigate the effects of H_2_ supplementation on CO-grown *C. autoethanogenum* chemostat cultures at the molecular level using metabolomics, proteomics, gas analysis, and a genome-scale metabolic model. We show that supply of H_2_ significantly reduces the carbon flux to CO_2_ and increases ethanol production. Proteomics data suggest that the metabolic rearrangements we observed are controlled at the post-translational level. We conclude that H_2_ supplementation can substantially improve the efficiency of gas fermentation and that H_2_ drives rearrangements at several molecular layers of acetogen metabolism.

## Results and discussion

### Gas-fermenting chemostat cultures of *Clostridium autoethanogenum*

*Clostridium autoethanogenum* is a promising biocatalyst for industrial-scale gas fermentation [4, 5], and therefore, it was used here to quantitate the effects of H_2_ supplementation on CO growth. Cells were grown on CO or CO + H_2_, termed “high-H_2_ CO” hereafter (H_2_/CO ~ 3), using a chemically defined medium in biological quadruplicate chemostats at dilution rate (D) ~ 1 day^−1^ (µ ~ 0.04 h^−1^). Two gas–liquid mass transfer rates were used resulting in steady-state biomass concentrations of ~ 0.5 and ~ 1.4 g dry cell weight (gDCW)/L. Cultures were subjected to gas analysis, metabolomics and proteomics (only ~ 1.4 gDCW/L) coupled with genome-scale metabolic modelling. We compare the data generated here to a previous syngas-grown data set (H_2_/CO ~ 0.4) [28] conducted at near-identical conditions (e.g., pH, D, biomass concentration).

### Supply of H_2_ leads to increased production of ethanol

**Fig. 1:**
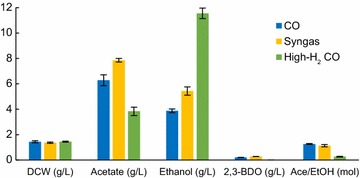
Steady-state by-product concentrations in gas-fermenting *C. autoethanogenum* chemostats. Data for high biomass concentration chemostats (~ 1.4 gDCW/L) are shown and represented as the average ± standard deviation between biological duplicates (syngas) and quadruplicates (CO and high-H_2_ CO). Syngas data from our previous work [28]. *DCW* dry cell weight, *Ace* acetate, *EtOH* ethanol, *2,3-BDO* 2R,3R-butanediol

Chemostats operated at two steady-state biomass concentrations provided insight into H_2_ supplementation at different levels of by-product inhibition, as we have previously shown that it can affect carbon distribution [28]. While acetate, ethanol, 2,3-butanediol (2,3-BDO), and traces of lactate have been detected in *C. autoethanogenum* batch cultures [31, 32], our continuous cultures showed production of all but lactate (Fig. 1 and Additional file 1: Fig. S1). Notably, increasing supply of H_2_ led to higher concentrations of ethanol (up to ~ 12 g/L) at similar biomass levels. The specific ethanol production rate (mmol/gDCW/h) was the highest in the low biomass high-H_2_ CO chemostats (Additional file 1: Fig. S2). Interestingly, acetate levels were the highest on syngas, while no 2,3-BDO was detected on high-H_2_ CO (Fig. 1 and Additional file 1: Fig. S1).

Previously, a molar acetate/ethanol ratio of ~ 1 was achieved in syngas-grown cells [28]. The high-H_2_ CO data presented here show that additional H_2_ supply can further direct carbon flux towards ethanol, achieving an acetate/ethanol ratios as low as ~ 0.2 (Fig. 1 and Additional file 1: Fig. S1). This highlights that the feed gas composition has a substantial effect on autotrophic metabolism and it is a critical factor for gas fermentation-based bioprocesses development, contributing to a reduction in downstream purification costs. In addition to fermentation parameters [26, 27, 33, 34] and genetic engineering tools used to enhance the biocatalysts [35–37], the composition of the feedstock gas is crucial to determine the economic performance of the bioprocess.

### Gas analysis shows that H_2_ uptake can substitute generation of redox from CO oxidation

**Fig. 2:**
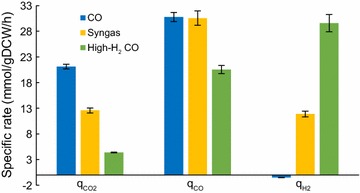
Steady-state gas uptake and production in gas-fermenting *C. autoethanogenum* chemostats. Data for high biomass concentration chemostats (~ 1.4 gDCW/L) are shown and represented as the average ± standard deviation between biological duplicates (syngas) and quadruplicates (CO and high-H_2_ CO). Syngas data from our previous work [28]. *DCW* dry cell weight, $q_{{{\text{CO}}_{2} }}$ specific CO_2_ production rate, *q*_CO_ and $q_{{{\text{H}}_{2} }}$ specific CO and H_2_ uptake rates, respectively

Analysis of gas consumption and production rates by an autotrophic culture is essential for understanding and comparing the flow of carbon and redox between cells grown on different gas mixes. Similar to the previous syngas-grown continuous cultures [26–28], we observed simultaneous utilisation of CO and H_2_ for both our H_2_-containing gas mixes during steady-state growth (Fig. 2 and Additional file 1: Fig. S3). Despite the high level of CO in the bioreactor off-gas for both the syngas and high-H_2_ CO high biomass cultures (~ 32 and ~ 9%, respectively), co-utilisation of CO and H_2_ was likely observed because of the relatively low residual CO concentration in the liquid phase, due to a CO-limited culture, in contrast to CO-excess batch cultures where co-utilisation is usually not observed [11, 15–17]. Importantly, higher supply of H_2_ enabled to more than double cellular H_2_ uptake (Fig. 2 and Additional file 1: Fig. S3). This was probably not caused by different levels of CO inhibition of hydrogenases, as the concentration of the chemostat-limiting substrate is determined by µ according to the Monod equation [38], and here, *µ* was near-constant across experiments (~ 0.04 h^−1^).

The specific CO_2_ production rate ($q_{{{\text{CO}}_{2} }}$) dropped more than fivefold with increasing H_2_ supply, while the specific CO uptake rate (*q*_CO_) was different between gases (high-H_2_ CO vs. others) only for the high biomass cultures (Fig. 2 and Additional file 1: Fig. S3). Notably, $q_{{{\text{CO}}_{2} }}$/*q*_CO_ significantly decreased with increasing H_2_ supply from 0.69 ± 0.01 to 0.21 ± 0.01 (average ± standard deviation) between high biomass CO and high-H_2_ CO cultures, respectively, indicating that less CO had to be dissipated as CO_2_ under H_2_ availability. This also suggests that lower generation of reducing power as reduced ferredoxin (Fd_red_) from the oxidation of CO into CO_2_ was off-set with increased H_2_ oxidation since both can serve as sources for the production of Fd_red_ [4, 11]. Indeed, $q_{{{\text{H}}_{2} }}$/*q*_CO_ increased around fourfold from 0.39 ± 0.01 to 1.44 ± 0.04 comparing high biomass high-H_2_ CO and syngas cultures. These observations confirm the potential of elevating cellular redox (and energy) generation by providing the culture with H_2_ [4, 5, 11], which is also shown by in silico metabolic modelling (see below).

### Carbon balance reveals that H_2_ supply substantially increases carbon flux to ethanol

**Fig. 3:**
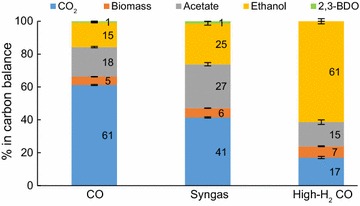
Carbon balances of gas-fermenting *C. autoethanogenum* chemostats. Carbon recoveries (refer to text) were normalised to 100% to have a fairer comparison of carbon distributions between the three gas mixes. Data for high biomass concentration chemostats (~ 1.4 gDCW/L) are shown and represented as the average ± standard deviation between biological duplicates (syngas) and quadruplicates (CO and high-H_2_ CO). Syngas data from our previous work [28]. *2,3-BDO* 2R,3R-butanediol

Coupling gas analysis with extracellular metabolomics in an autotrophic continuous culture enables accurate carbon balancing, which allows experimental validation of theoretical stoichiometric calculations of product yields [5, 11, 14]. Our high biomass steady-state cultures showed carbon recoveries of 111 ± 2, 99 ± 2, and 124 ± 2% for CO, syngas, and high-H_2_ CO, respectively (see Additional file 1: Fig. S4 for low biomass data). Carbon recoveries were normalised to 100% to have a fairer comparison of carbon distributions between the three gas mixes. Most importantly, H_2_ supplementation realised a fourfold higher carbon flux to ethanol (15 ± 0.2% vs. 61 ± 2%) due to the proportional decline of carbon loss as CO_2_ (61 ± 0.3% vs. 17 ± 1%) when comparing high biomass CO and high-H_2_ CO cultures (Fig. 3). Even more significant effects were seen for low biomass cultures (Additional file 1: Fig. S4). Thus, the supply of H_2_ can indeed significantly diminish the loss of CO as CO_2_ and boost carbon flux to ethanol, as estimated by theoretical stoichiometric calculations [14].

**Fig. 4:**
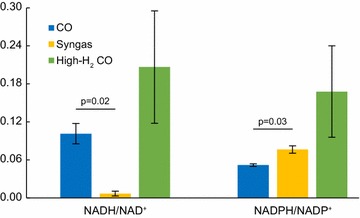
Intracellular redox state of gas-fermenting *C. autoethanogenum* chemostats. Data for high biomass concentration chemostats (~ 1.4 gDCW/L) are shown and represented as the average ± standard deviation between biological triplicates (syngas and high-H_2_ CO NADH/NAD^+^) and quadruplicates (CO and high-H_2_ CO NADPH/NADP^+^). Syngas data from our previous work [28]. The *p* values are calculated using a paired two-tailed *t* test

Though higher production of reduced by-products leads to a more oxidised intracellular redox state inferred from NADH/NAD^+^ and NADPH/NADP^+^ measurements [28, 39, 40], supply of H_2_, however, may result in a more reduced redox state. Comparing our syngas cultures with supply of H_2_ and higher carbon flux to reduced by-products compared to CO (Fig. 3), intracellular metabolome analysis of 33 metabolites (Additional File 2: Tables S1, S2) showed a lower NADH/NAD^+^ (*p* value = 0.02, paired two-tailed *t* test) but a higher NADPH/NADP^+^ (*p* value = 0.03) in syngas compared to CO (Fig. 4). However, no statistically significant differences (*p* value < 0.05) in redox ratios were observed between high-H_2_ CO and CO or syngas (Fig. 4), suggesting that H_2_ supply might not necessarily contribute to a more reduced cellular redox state.

Based on the stoichiometries for ethanol synthesis from CO reported by Wilkins and Atiyeh [14], ~ 67 or ~ 17% of CO is lost as CO_2_ if no H_2_ is supplied or H_2_/CO ~ 1.5, respectively. Although our cultures also produced acetate, biomass, and 2,3-BDO, the estimates of Wilkins and Atiyeh [14] are very close to our experimental data: 61% of carbon lost as CO_2_ in CO cultures and 17% of carbon lost as CO_2_ in high-H_2_ CO cultures with $q_{{{\text{H}}_{2} }}$/*q*_CO_ ~ 1.4 (high biomass data). This might be somewhat surprising, since the conversion of CO to ethanol with higher H_2_ utilisation becomes thermodynamically less favourable due to the lower Gibbs free energy change [14], which, however, does not seem to be a problem for the cells. We chose a feed gas H_2_/CO of ~ 3 for the high-H_2_ CO cultures as theoretical stoichiometric calculations estimate no carbon loss as CO_2_ above an H_2_/CO uptake ratio of two during ethanol formation from CO [14]. Further process optimisation is needed for achieving such a high uptake ratio to test whether carbon loss as CO_2_ could be eliminated.

It is noteworthy that incorporation of carbon into biomass (i.e., biomass yield) increased by ~ 35% (5.1 ± 0.1 vs. 6.8 ± 0.3%) with increasing H_2_ supply (Fig. 3). Carbon flux into acetate dropped by ~ 22% (18 ± 0.4 vs. 15 ± 1.3%; *p* value = 0.003) when comparing high biomass cultures of high-H_2_ CO to CO. Our data show that H_2_ supplementation improves the efficiency of ethanol production using gas fermentation through higher substrate fluxes into ethanol and biomass with decreased loss of carbon into other by-products.

### Analysis of the effects of H_2_ on intracellular metabolism using a genome-scale metabolic model

Comprehensive quantification of carbon and redox flows in and out of the cells enables the estimation of intracellular flux patterns using genome-scale metabolic models (GEMs) [41, 42]. Our chemostats provided steady-state data, which are preferred for accurate in silico reconstruction of metabolism. We used the GEM iCLAU786 previously developed for *C. autoethanogenum* [43] for in silico analysis of metabolism (refer to Methods for details). Simulation results identified as SIMx (e.g., SIM1) in the text are reported in Additional file 3: Tables S3, S4. The impact of H_2_ supply on intracellular fluxes was estimated by constraining the GEM with experimental data (exchange rates and *µ*) and maximising dissipation of ATP as the objective function in flux balance analysis (FBA) [44] calculations (SIM1–19). Only high biomass data are discussed here, since similar observations were made for low biomass conditions (Additional file 1: Fig. S5).

**Fig. 5:**
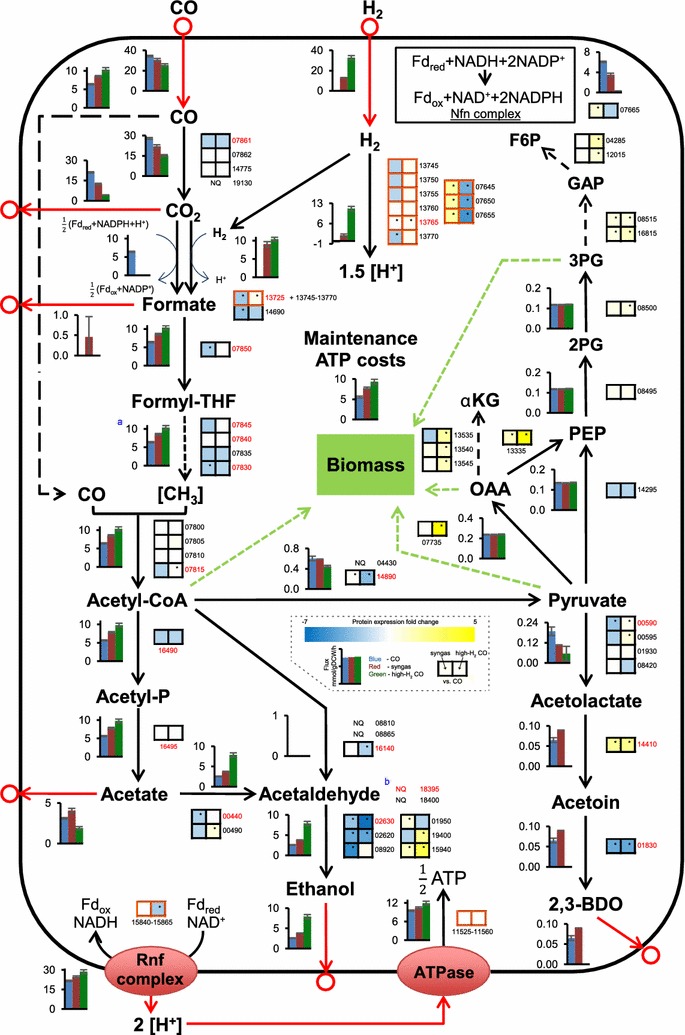
Central metabolism flux levels and relative protein expression of high biomass gas-fermenting *C. autoethanogenum* chemostats. Data for high biomass concentration chemostats (~ 1.4 gDCW/L) are shown. See dashed inset for bar chart and heatmap details. Fluxes (mmol/gDCW/h) are represented as the average ± standard deviation between duplicate (syngas) and quadruplicate (CO and high-H_2_ CO) chemostats. Arrows show the direction of calculated fluxes; red arrow denotes uptake or secretion. Flux into PEP from OAA and pyruvate is merged. Refer to Additional file 1: Fig. S5 for the cofactors of the reactions used in the model and Additional file 2: Table S2 for metabolite abbreviations. Protein expression fold changes are average of quadruplicate chemostats: syngas vs. CO (left box) and high-H_2_ CO vs. CO (right box). SIL-protein-aided label-based data are denoted with red font for gene ID. Differentially expressed proteins are indicated with an asterisk (*q* value < 0.05 after false discovery rate [FDR] correction [58], and for label-free data additionally fold-change > 1.5). Proteins forming a complex are highlighted with orange borders; FdhA (13725) forms a complex with HytA–E (13745–13770) for direct CO_2_ reduction with H_2_. Median data are shown for the Rnf and ATPase protein complexes. ^a^Methylene-THF reductase flux is shown; ^b^bifunctional acetaldehyde/alcohol dehydrogenase (acetyl-CoA → ethanol). Gene IDs next to heatmaps are preceded with CAETHG_RS; *gDCW* gram dry cell weight, *NQ* not quantified. Refer to Additional file 3: Tables S3 and S4 for flux data and Additional file 4: Tables S5–S7 for protein expression data

Simulations showed that, although CO oxidation declined with increasing H_2_ supply, flux through the WLP increased due to the lower dissipation of CO_2_ from CO oxidation (Fig. 5). A higher flux through the WLP demands both more ATP and reducing power (see Additional file 1: Fig. S5 for central metabolism cofactors used in the model). While the redox for this was supplied directly by the significantly elevated H_2_ oxidation (~ 82% vs. ~ 41% of Fd_red_ produced by CO oxidation on CO vs. high-H_2_ CO), it also enabled higher ATP production by providing Fd_red_ to the Rnf–ATPase system (Additional file 3: Table S3). Notably, these changes were accompanied by the complete shutdown of flux through the Nfn transhydrogenase complex (Fig. 5). In addition to the use of H_2_ for redox generation, all the CO_2_ fixed by the WLP was reduced to formate directly using H_2_ by the formate-H_2_ lyase activity of the electron-bifurcating hydrogenase–formate dehydrogenase (HytA–E/FdhA) enzyme complex [18] (Fig. 5). This saves redox compared to growth on CO, where the redox-consuming formate dehydrogenase catalysed the reduction of CO_2_. These observations show that cells have the flexibility to use the extra H_2_ left over from CO_2_ reduction for carbon redistribution (Fig. 3).

Simulations of CO and high-H_2_ CO conditions confirmed that ethanol is produced solely through acetate using the acetaldehyde:ferredoxin oxidoreductase (AOR) activity, rather than through the “conventional” direct route from AcCoA using the bifunctional acetaldehyde/alcohol dehydrogenase AdhE activity (Fig. 5), as previously reported for syngas [28]. Our proteomics data (see below) also show much higher expression levels for AOR (CAETHG_RS00440) compared to genes of the “conventional” route as previously shown by transcript abundance measurements in *C. autoethanogenum* syngas cultures [28] and by other acetogen data sets [45–47]. The dominance of AOR is not surprising as it enables coupling ethanol and ATP production, especially important during growth on CO_2_ and H_2_ [11, 48]. Our calculations with CO and high-H_2_ CO data agree with the result of syngas data [28] that the final WLP reduction step catalysed by methylene-THF reductase (MetFV, RS07830, and RS07835) is most likely Fd reducing, since in silico growth was infeasible if the latter activity was not present. These results are important for filling gaps in our understanding and mathematical description of energy conservation in acetogens [48, 49].

We detected an increase in cellular maintenance costs [50, 51] from 5.5 ± 0.2 to 9.3 ± 0.6 mmol/gDCW/h with increasing H_2_ supply (Fig. 5). Interestingly, the fraction of maintenance costs from total ATP production also increased, from ~ 37 to ~ 43% (Additional file 3: Table S3). The higher ATP demands for cellular maintenance in high-H_2_ CO cultures could be explained by the chaotropic nature of ethanol (~ 12 g/L) causing the leakage of protons from compromised cell membrane integrity [52] and significant consumption of H_2_ as both lead to the influx of protons without ATP synthesis (the Rnf-ATPase system in *C. autoethanogenum* generates ATP through proton motive force [53, 54]).

### Prediction of “optimal” growth phenotypes using a genome-scale metabolic model

In addition to estimating intracellular flux patterns, GEMs are useful for phenotype prediction, i.e., designing strains in silico, with potentially superior characteristics (e.g., higher target product yield) [41, 42]. Before using the model for strain design, it is useful to evaluate the accuracy of the model by predicting “optimal” growth phenotypes. This is generally done by constraining the model with experimental data from substrate uptake rates and maintenance ATP costs and by maximising biomass yield in FBA calculations.

Similar to our previous predictions for syngas without additional constraints [28], neither ethanol nor 2,3-BDO production were predicted by the model for “optimal” growth on CO, while ethanol production on high-H_2_ CO was underestimated by ~ 55% (Additional file 3: Table S3 SIM20–34). We discovered in the former work [28] that coupling of carbon and redox metabolism from H_2_ utilisation enables the GEM to predict ethanol close to experimental values. Indeed, ethanol prediction on high-H_2_ CO was improved with the extra constraint (see “Methods” for details) to differ by ~ 4% from experimental values (Additional file 1: Fig. S6; Additional file 3: Table S3 SIM35–41). Despite the latter and the ability to accurately predict growth-boosting amino acids for *C. autoethanogenum* [43], in silico reconstruction of autotrophic growth is still incomplete as ethanol and 2,3-BDO production on CO could not be predicted. The most likely shortcoming of using stoichiometric-only models (e.g., a GEM) for predicting “optimal” phenotypes is not taking into account the effects of the high concentrations of ethanol and acetate on enzyme kinetics and cellular maintenance costs, highlighting the need for kinetic models [41].

### Proteome analysis showed no obvious changes explaining the metabolic rearrangements

We next performed quantitative proteome analyses to investigate whether the above-described metabolic rearrangements could be explained by changes in intracellular protein levels for high biomass cultures of the three gas mixes. We used a data independent acquisition (DIA) mass spectrometry approach [55] to confidently quantitate 1655 proteins across and 1403 in average within each sample (12 in total) with at least two peptides per protein. Our proteomics data were highly reproducible shown by the clear clustering of bio-replicates with an average Pearson correlation coefficient of *R* = 0.98 between them (Additional file 1: Fig. S7A–C). To increase the accuracy of relative protein quantification, 20 stable-isotope labelled (SIL) proteins covering central metabolism, the HytA–E hydrogenase, and a ribosomal protein of *C. autoethanogenum* (Additional file 4: Table S5) were synthesised using a wheat germ cell-free system [56, 57] and spiked into every sample as SIL peptides.

While the proteomics data showed gas mix-specific clustering (Additional file 1: Fig. S7D) and ~ 200 proteins were differentially expressed (fold-change > 1.5, *q* value < 0.05 after false discovery rate [FDR] correction [58]) between syngas and CO (Additional file 4: Table S6) or high-H_2_ CO and CO (Additional file 4: Table S7) in the label-free proteome-wide data set, no obvious protein expression changes were observed explaining the metabolic rearrangements (Fig. 5). In addition, the SIL-protein-aided label-based relative quantification did not provide clear answers for this (Fig. 5 and Additional file 4: Table S5). Among the 13 proteins associated with ethanol synthesis from either acetate or acetyl-CoA, only one—an alcohol dehydrogenase (RS15940)—showed consistent up-regulation with increased flux to ethanol (Fig. 5). Importantly, the primary AOR (RS00440) seems to facilitate ethanol synthesis as it is a top-10 protein by absolute expression levels (MS signal intensities), and none of the mono- or bifunctional acetaldehyde/alcohol dehydrogenases of the “conventional” route directly from acetyl-CoA were quantifiable (Additional file 4: Table S8 and Fig. 5). This is consistent with our model simulations showing flux only through AOR (Fig. 5). Regarding the acetaldehyde-to-ethanol step, despite RS15940 showing consistent up-regulation, RS08920 is likely the most relevant alcohol dehydrogenase as its absolute expression levels greatly surpassed the other five alcohol dehydrogenases (25–500-fold in high-H_2_ CO). It is important to note that fluxes of by-product synthesis pathways—ethanol, acetate, and 2,3-BDO—seem to be regulated post-translationally as protein expression did not follow changes in flux rates (Fig. 5).

One would expect the expression of cellular hydrogenases to increase with elevated H_2_ supply and uptake, which increased from sub-zero (i.e., minor production) on CO to ~ 30 mmol/gDCW/h on high-H_2_ CO (Figs. 2, 4). Strikingly, *C. autoethanogenum* hydrogenases seem to be expressed constantly within our three gas mixes at high enough levels to realise substantially elevated H_2_ utilisation as neither of the two quantifiable hydrogenase complexes—HytA–E (RS13745–13770) and another iron-containing complex (RS07645–07655)—from the six annotated in the genome [59] showed a higher than threefold increase in protein expression (Fig. 5). The opposite expression dynamics of these two hydrogenases—down- and up-regulation, and up- and down-regulation of HytA and RS07645–07655 when comparing syngas to CO and high-H_2_ CO to CO, respectively—could suggest different optimal operation conditions (Fig. 5 and Additional file 4: Tables S5–S7). Both hydrogenases show high absolute expression levels (Additional file 4: Table S8), as previously seen in *C. autoethanogenum* RNA sequencing data sets [28, 45, 59]. High expression of hydrogenases, even on CO, could be explained by the capability of acetogens to rapidly catabolise H_2_ once it becomes available in natural environments, thus providing a growth advantage.

Our model simulations showed that for H_2_-containing gas mixes all the CO_2_ fixed by the WLP was reduced to formate directly using H_2_ by the formate-H_2_ lyase activity of the HytA–E/FdhA enzyme complex (Fig. 5). Proteomics data support this as the expression profile of HytA and FdhA was very close (all changes *q* value < 0.05; Fig. 5 and Additional File 4: Tables S6, S7), and the only other quantifiable formate dehydrogenase (RS14690) showed very low absolute expression levels (Additional file 4: Table S8). Notably, concomitant protein expression changes with increasing flux through the WLP were not observed (Fig. 5), similar to hydrogenases’ expression suggesting that sufficient enzymatic capacity was constantly expressed. Overall, our proteomics data suggest that fluxes of acetogen central metabolism are regulated post-translationally, as proposed before [47], which further highlights the need for the use of kinetic models for more accurate reconstruction of acetogen metabolism in silico [41]. Further analyses are needed to determine which mechanism from allosteric regulation, substrate concentration change or post-translational protein modification is responsible for post-translational regulation of fluxes.

There were other notable differentially expressed proteins within the pool of ~ 200. For example, proteins belonging to the pyruvate–oxaloacetate–PEP node (RS07735 and RS13335; both ~ fivefold, *q* < 0.01) and several gluconeogenetic enzymes were up-regulated on high-H_2_ CO compared to CO (Fig. 5). In addition, expression of proteins of the UMP biosynthesis pathway (RS07105–07130; in average ~ threefold, *q* < 0.01) and its associated glutamate–glutamine metabolic pathways (in average ~ twofold, *q* < 0.01), and of the oxidative TCA cycle (RS13535–13545; in average ~ twofold, *q* < 0.01) were elevated on high-H_2_ CO compared to CO (Additional file 4: Table S7 and Fig. 5). While the intracellular UMP metabolite concentration was not higher in the latter condition, concentrations of UDP and UTP, downstream products of UMP, were higher on high-H_2_ CO (Additional file 2: Table S1).

## Conclusions

Our results show that feed gas composition has a strong effect on autotrophic metabolism, particularly the presence and level of H_2_. We conclude that H_2_ supplementation can substantially improve the efficiency of gas fermentation by realising higher substrate fluxes into ethanol and biomass with decreased loss of carbon into other by-products. Proteomics data suggest that the observed metabolic rearrangements were regulated at the post-translational level. Our study, representing the first quantitative analysis of the effects of H_2_ supplementation on CO-grown acetogens under controlled growth conditions, provides novel links between carbon, energy, and redox metabolism. Altogether, the data presented here advance our understanding of energy conservation in acetogens [48, 49]. The results also highlight that feed gas composition can be considered a critical factor in enhancing the economics of gas fermentation-based bioprocesses.

## Methods

### Bacterial strain, growth medium, and continuous culture conditions

A derivate of the *Clostridium autoethanogenum* DSM 10061 strain—DSM 19630—deposited in The German Collection of Microorganisms and Cell Cultures (DSMZ) was used in all experiments and stored as glycerol stocks at − 80 °C.

Cells were grown either on CO (~ 60% CO and 40% Ar; BOC Australia) or CO + H_2_, termed “high-H_2_ CO” here (~ 15% CO, 45% H_2_ and 40% Ar; BOC Australia) in chemically defined medium (without yeast extract) [28]. Experimental data for growth on syngas (~ 50% CO, 20% H_2_, 20% CO_2,_ and 10% N_2_/Ar; BOC Australia) other than proteomics data, which was generated in this work, are from our previous study [28].

As during growth on syngas [28], cells were grown under strict anaerobic conditions at 37 °C and at a pH of 5 (maintained by 5 M NH_4_OH). Chemostat continuous cultures were operated at D = 1.0 ± 0.01 day^−1^ (µ = 0.04 ± 0.001 h^−1^) and D = 1.0 ± 0.01 day^−1^ (µ = 0.04 ± 0.001 h^−1^) for CO and high-H_2_ CO, respectively, (D = 1.0 ± 0.03 day^−1^ [µ = 0.04 ± 0.001 h^−1^] for syngas) in 1.4 L Multifors bioreactors (Infors AG) at a working volume of 750 mL. The system was equipped with peristaltic pumps; mass flow controllers (MFCs); pH, ORP, and temperature sensors and was connected to a Hiden HPR-20-QIC mass spectrometer (Hiden Analytical) for online high-resolution off-gas analysis. Antifoam was continuously added to the bioreactor using a syringe pump to avoid foaming.

We targeted the lowest and highest (~ 0.5 and 1.4 gDCW/L, respectively) steady-state biomass concentrations of the previous syngas cultures [28] to compare the effect of H_2_ supplementation at different levels of inhibition from by-products. This was achieved using various steady-state gas–liquid mass transfer rates: for CO, 510 and 665 RPM at 46.5 mL/min gas flow resulting in 0.47 ± 0.02 and 1.43 ± 0.08 (gDCW/L), respectively; for high-H_2_ CO, 650 and 1000 RPM, and 46.5 and 110 mL/min gas flow resulting in 0.46 ± 0.04 and 1.45 ± 0.04 (gDCW/L), respectively. Four biological replicate cultures with two steady states (low and high biomass) per independent chemostat run were performed. All the steady-state results reported here were collected after optical density (OD) and gas uptake and production rates had been stable in chemostat mode for 3–5 working volumes, similar to syngas data.

### Biomass concentration analysis

Biomass concentration (gDCW/L) was estimated for CO and high-H_2_ CO cultures by measuring the OD of the culture at 600 nm using the correlation coefficient of 0.21 between culture OD and dry cell weight determined in [28] for syngas cultures.

### Bioreactor off-gas analysis

Bioreactor off-gas analysis was performed as specified in [28] by an online Hiden HPR-20-QIC mass spectrometer (MS) using the Faraday Cup detector. Shortly, gas uptake (CO and H_2_) and production (CO_2_ and ethanol) were determined using “online calibration” of the MS by analysing the respective feed gas directly from the cylinder after each analysis cycle of the bioreactors. Specific rates (mmol/gDCW/h) were calculated by taking into account the exact composition of the respective gas, bioreactor liquid working volume, feeding gas flow rate, off-gas flow rate based on the fractional difference of the inert gas Ar in the feeding and off-gas composition, the molar volume of ideal gas, and the steady-state biomass concentration. To achieve a more accurate carbon balance, ethanol stripping and the total soluble CO_2_ fraction in culture broth were also taken into account based on off-gas analysis.

### Extracellular metabolome analysis

Extracellular metabolome analysis was carried out using filtered broth samples stored at − 20 °C until analysis. Organic acids, alcohols, and amino acids were quantified using HPLC as described before [43]. We note that cells produced 2R,3R-butanediol.

### Intracellular metabolome analysis

Intracellular metabolome analysis was based on the method previously developed for the autotrophic growth of *C. autoethanogenum* [45] with details specified in [28]. Briefly, 1 mL of a high biomass culture was pelleted by immediate centrifugation followed by extraction of intracellular metabolites using acetonitrile. Metabolite concentrations were determined using LC–MS analysis in negative ion mode and relevant standards.

### Cell-free synthesis of stable-isotope labelled proteins

Twenty proteins covering central metabolism, the HytA–E hydrogenase, and a ribosomal protein of *C. autoethanogenum* (Additional file 4: Table S5) were selected for cell-free synthesis of stable-isotope labelled (SIL) proteins. First, the genes encoding for these proteins were synthesised by commercial gene synthesis services (Biomatik). The PCR-amplified target genes were sub-cloned into the cell-free expression vector pEUE01-His-N2 (Cell-Free Sciences) and transformed into *Escherichia coli* DH5α. Next, plasmid DNA was extracted and purified by alkaline lysis after cells had been cultured overnight in LB medium containing 50 μg/mL ampicillin. Correct gene insertion into the pEUE01-His-N2 was verified by DNA sequencing. Subsequently, cell-free synthesis of His-tag fused *C. autoethanogenum* proteins was performed using the bilayer reaction method with the wheat germ extract WEPRO8240H (Cell-Free Sciences) as described previously [56, 57]. Briefly, mRNAs used for cell-free synthesis were prepared by an in vitro transcription reaction at 37 °C for 6 h using the SP6 RNA polymerase. In vitro translation of *C. autoethanogenum* proteins was performed using a bilayer reaction (200 μL substrate layer and 40 μL translation layer) at 17 °C for 24 h in a 96-well microplate. The translation layer was supplemented with l-Arg-^13^C_6_,^15^N_4_ and  l-Lys-^13^C_6_,^15^N_2_ (Wako) at final concentrations of 20 mM to achieve high efficiency (> 99%) stable-isotope labelling of proteins. Finally, in vitro synthesised proteins were purified using the Ni-Sepharose High-Performance resin (GE Healthcare Life Sciences) and stored at − 80 °C until further use.

### Proteome analysis

Proteome analysis of CO, high-H_2_ CO, and syngas cultures was carried out for four biological replicates from the high biomass concentration (~ 1.4 gDCW/L) experiments using a DIA MS approach [55]. 2 mL of culture was pelleted by immediate centrifugation (25,000×*g* for 1 min at 4 °C) and stored at − 80 °C until analysis.

#### Sample preparation

Frozen cell pellets were thawed, washed with phosphate-buffered saline, resuspended in 500 µL of lysis buffer (pH 7.6) containing 2% (w/v) SDS (L4390; Sigma-Aldrich), 0.1 M DTT (V3155; Promega), 0.1 M Trizma^®^ base (T1503; Sigma-Aldrich), and vortexed. The cell suspension was transferred to a 2 mL screw cap microtube (522-Q; Thermo Fisher Scientific) containing 0.1 mm glass beads (11079101; BioSpec Products). Cell lysis was performed by repeating the following “lysis cycle” four times: incubation for 10 min at 100 °C; bead beating using program “cycle 5” on the Precellys™ 24 instrument (Bertin Technologies); centrifugation at 14,000 rpm for 10 min at room temperature; vortexing (excluded from the final fourth lysis cycle). Next, 400 µL of lysate was carefully removed without withdrawing glass beads. Protein concentration in cell lysates was determined using the 2D Quant Kit (80-6483-56; GE Healthcare Life Sciences).

Alkylation of sulfhydryl groups and protein digestion was based on the filter-aided sample preparation (FASP) protocol [60]. 100 µg of protein was loaded and mixed with 200 µL of 8 M urea ([UA]; U5128; Sigma-Aldrich) in 0.1 M Trizma^®^ base (pH 8.5) on an Amicon^®^ Ultra-0.5 mL centrifugal filter unit with nominal molecular weight cutoff of 30,000 (UFC503096; Merck Millipore), and centrifuged at 14,000 rpm for 10 min at room temperature. The filter was washed and centrifuged once more with 200 µL of UA after which sulfhydryl groups were alkylated with the addition of 100 µL of 0.05 M iodoacetamide (I6125; Sigma-Aldrich) in UA, vigorous vortexing, and incubation for 30 min at room temperature in the dark. Next, the filter was centrifuged as described above, and then washed three times and centrifuged with UA. Subsequently, the addition of 100 µL of 25 mM ammonium bicarbonate (AMBIC) and centrifugation was repeated twice before proteins were digested on the filter for 16 h at 37 °C with 2 µg of Trypsin/Lys-C mix (V5073; Promega) in 30 µL of ~ 17 mM AMBIC and acetic acid. Peptides were recovered by centrifuging the filters upside down at 1000 rpm for 2 min at room temperature, followed by two times of addition of 30 µL of 25 mM AMBIC and centrifugation as in the previous step. Finally, the collected peptide material was mixed with 10 µL of 0.1% (v/v) formic acid (FA) in 5% (v/v) acetonitrile (ACN) to stop digestion.

Samples were desalted using C_18_ ZipTips (ZTC18S096; Merck Millipore) as follows: the column was wetted using 0.1% FA in 100% ACN, equilibrated with 0.1% FA in 70% (v/v) ACN, and washed with 0.1% FA before loading the sample and washing again with 0.1% FA. Finally, peptides were eluted with 0.1% FA in 70% ACN. Total peptide concentration in each sample was determined using the Pierce™ Quantitative Fluorometric Peptide Assay (23290; Thermo Fisher Scientific) to ensure that the same total peptide amount across samples could be injected for DIA MS analysis. To further increase the accuracy of relative protein quantification, each sample was spiked with the same amount of a mix of SIL peptides derived from the 20 SIL proteins (see above) using the same FASP-based workflow as for culture samples with an additional step of reduction of disulfide bonds using DTT. Finally, samples were freeze-dried and reconstituted with 10 µL of 2% (v/v) ACN containing 0.05% (v/v) trifluoroacetic acid (TFA) to which 1 µL of an iRT Peptide mix (Ki-3003; Biognosis) were added, pre-diluted one in five to meet the manufacturer’s recommendations. In addition, the whole material eluted from a ZipTip of one sample from each gas mix and one syngas-grown culture sample spiked with SIL peptides were used for DIA MS spectral library generation using data-dependent acquisition (DDA; see below).

#### Sample fractionation for DIA MS spectral library

To increase the proteome coverage in DIA MS analysis, a pool of samples from each gas mix was fractionated using high pH reverse-phase fractionation, based on the protocol of the Thermo Fisher Scientific product 84868. A Waters Sep-Pak tC18 cartridge (WAT054960; Waters) was conditioned twice by the addition of 500 µL of 100% ACN and centrifugation inside a 15 mL falcon tube at 3000×*g* for 2 min at room temperature. The same was repeated with 0.1% FA. Next, ~ 15 µg of the FASP product of one sample from each gas mix were pooled together, mixed with 0.1% FA for a final volume of 500 µL and loaded on the column by centrifugation (same conditions). The cartridge was then washed with 500 µL of Milli-Q water before eight fractions were collected with an increasing ACN step gradient (from 5 to 50%) at high pH in triethylamine background. Finally, the fractions were freeze-dried and reconstituted with 10 µL of 2% ACN containing 0.05% TFA.

#### Nano-LC method

For both the DDA spectral library generation and DIA sample runs, a Thermo-Scientific U3000 nano-HPLC system was used in a trap column configuration for concentration and separation of the peptide samples. The samples were initially loaded onto a Thermo Acclaim PepMap C_18_ trap reversed-phase column (75 µm × 2 cm nano viper, 3 µm particle size) at a flow rate of 8 µL/min using 2% ACN containing 0.05% TFA for 6 min. Separation was achieved at 250 nL/min using 0.1% FA in water (buffer A) and 0.1% FA in ACN (buffer B) as mobile phases for gradient elution with a 75 µm × 50 cm PepMap RSLC C_18_ (2 µm particle size) Easy-Spray Column at 45 °C. Peptide elution employed a 2–8% ACN gradient for 14 min followed by two step gradients of 8–30% ACN gradient for 80 min and 30–45% ACN for 10 min. The total acquisition time was 130 min including a 95% ACN wash and re-equilibration step. For each DDA sample run, a volume of 5 µL equating to ~ 1.5 µg of protein digest was injected. Likewise, for each DIA sample run, a volume of 5 µL equating to 0.5 µg of protein digest was injected.

#### DIA MS spectral library generation

The following 17 samples were analysed on the Q-Exactive HF (Thermo Fisher Scientific) in DDA mode to yield the spectral library for DIA MS data analysis: (1) one unfractionated sample from each gas mix and one unfractionated syngas-grown culture sample spiked with SIL peptides; (2) four replicates of one pool of all 12 unfractionated culture samples; (3) eight high pH reverse-phase fractions of a pool of samples from each gas mix; and (4) a mix of eight SIL-proteins (see Additional file 4: Table S5).

The eluted peptides from the C_18_ column were introduced to the MS via a nano-ESI and analysed using the Q-Exactive HF. The electrospray voltage was 1.8 kV, and the ion transfer tube temperature was 250 °C. Employing a top-20 ddMS2 acquisition method, full MS scans were acquired in the Orbitrap mass analyzer over the range *m/z* 400–1200 with a mass resolution of 120,000 (at *m/z* 200). The AGC target value was set at 3.00E+06. The 20 most intense peaks with a charge state between 2 and 6 were fragmented in the high energy collision dissociation (HCD) cell with a normalised collision energy of 28%. MSMS spectra were acquired in the Orbitrap mass analyzer with a mass resolution of 15,000 at *m/z* 200. The AGC target value for MSMS was set to 1.0E+05, while the ion selection threshold was set to 1.8E+05 counts. The maximum allowed ion accumulation times were 50 ms for full MS scans and 40 ms for MSMS. For all the experiments, the dynamic exclusion time was set to 20 s, and undetermined charge state species were excluded from MSMS.

Identification results from DDA analysis were used to build a spectral library for DIA MS data confirmation and quantification using Skyline [61] (see below). For this, raw DDA data files were analysed with Proteome Discoverer 2.2 (Thermo Fisher Scientific) using SEQUEST HT against a *C. autoethanogenum* DSM 10061 [31] database containing ~ 3750 sequences while also annotated to include the 20 SIL-proteins and a fusion of the 11 iRT peptides. The NCBI annotation of sequence NC_022592.1 [59] was used as the annotation genome here, with CAETHG_RS07860 removed and replaced with the carbon monoxide dehydrogenase genes named CAETHG_RS07861 and CAETHG_RS07862 with initial IDs of CAETHG_1620 and 1621 [59], respectively. The workflow editor was used to create customised searches and result reports, where RAW data files were processed to generate a Magellan Server File (MSF) result file and a.pd result output file, which was later incorporated in Skyline for the DIA MS spectral library build.

The SEQUEST HT search parameters included: 10 ppm precursor ion mass tolerance; product ion mass tolerance of 0.05 *m/z*; full trypsin specificity with two missed cleavages allowed for peptides with a length of 6–150 AAs. Cysteine carbamidomethylation was set as a fixed modification, while methionine oxidation, deamidation of glutamine and asparagine as well as N-terminal acetylation were set as variable modifications. The mass analyser used was Fourier Transform Mass Spectrometry while the activation type was HCD. Peaks were filtered with a signal to noise (S/N) threshold of 1.5. A separate SEQUEST HT search node was included with fixed modifications set to include ^13^C(6)^15^N(2)/+ 8.014 Da (*K*) and ^13^C(6)^15^N(4)/+ 10.008 Da (*R*) for the SIL-proteins. Within this search node, cysteine carbamidomethylation (+ 57) was set as a fixed modification, while methionine oxidation (+ 16), deamidation of glutamine and asparagine (+ 0.984) as well as N-terminal acetylation (+ 42) were set as variable modifications.

Database searching against the corresponding decoy database containing reversed protein sequences was performed using Percolator to evaluate the FDR of peptide identifications. The final.pd result file contained peptide–spectrum matches (PSMs) with *q* values estimated at 1% FDR for peptides ≥ 4 AAs.

#### DIA MS data acquisition

As for the DDA method, eluted peptides were introduced to the MS via a nano-ESI and analysed using the Q-Exactive HF. The electrospray voltage was 1.9 kV, and the ion transfer tube temperature was 250 °C. DIA was achieved using an inclusion list: *m/z* 400‒1000 in steps of 15 amu and a quadruple isolation window of 16 amu, scans cycled through the list of 40 isolation windows interspersed with an MS1 scan for every 10 targets. Full MS scans were acquired in the Orbitrap mass analyser over the range *m/z* 400–1200 with a mass resolution of 120,000 (at *m/z* 200). Identical to the DDA method, the AGC target value was set at 3.00E+06 with a maximum injection time of 50 ms. All DIA scans implemented an NCE collision energy of 28% while MSMS detection in the Orbitrap was at a resolution setting of 30,000 (at *m/z* 200). The AGC target was set to 1.0E+06 with a maximum injection time of 45 ms. A first fixed mass of *m/z* 200 was applied, and default charge state of 2 was set for scanning MS2 events.

#### DIA MS data analysis

DIA MS data analysis was performed with the software Skyline [61]. The .pd result file from Proteome Discoverer was used to build the DIA MS spectral library within Skyline using only PSMs with *q* value < 0.01. The following parameters were used for DIA MS data analysis: six of the most intense y and b (only y for SIL-protein-aided label-based quantification) product ions from ion 3 to last ion of charge state 1 and 2 among precursor charges 2, 3, and 4 were picked while product ions falling within the DIA precursor window were excluded; chromatograms were extracted with a library match mass tolerance of 0.05 *m/z* for product ions with an extraction window within 5 min of the predicted retention time after iRT alignment; full trypsin specificity with two missed cleavages allowed for peptides with a length of 8–25 AAs; cysteine carbamidomethylation as a fixed peptide modification. In addition, for SIL-protein-aided label-based quantification, peptide modifications included heavy labels for lysine and arginine as ^13^C(6)^15^N(2)/+ 8.014 Da (*K*) and ^13^C(6)^15^N(4)/+ 10.008 Da (*R*), respectively, and these heavy labels were set as “internal standard type” to aid peak picking.

Next, for both data sets (label-free and label-based), shared peptides were removed, and a minimum of five transitions per precursor and two peptides per protein were allowed. The mProphet peak picking algorithm [62] within Skyline was used and trained with shuffled sequence decoys to separate true from false-positive peak groups (per sample) and only peak groups with *q* value < 0.01 (representing 1% FDR) were used for further quantification. For the proteome-wide data set (label-free), we confidently quantitated 14,705 peptides and 1655 proteins across all samples, and 10,134 peptides and 1403 proteins on average within each sample with at least two peptides per protein.

#### Determination of differentially expressed proteins and absolute protein expression levels

Protein expression fold changes with *p*- and *q* values were determined using the software MSstats [63] with high-quality feature selection, top3 featureSubset, and Tukey’s median polish as run-level summarisation within its linear mixed models. For the proteome-wide data set (label-free), only proteins with at least two peptides in each bio-replicate under comparison were used (filtering for two, instead of one, peptides per protein has shown higher quantification accuracy [64, 65]) and input data were normalised using quantile normalisation, independently determined as the most suitable normalisation method using the software Normalyzer [66]. Higher quantification accuracy for the SIL proteins was achieved by label-based quantification through normalising light (endogenous) data with heavy (spike-in). Proteins were considered to be differentially expressed by a *q* value < 0.05 after FDR correction [58], and for proteome-wide label-free quantification additionally with a fold-change > 1.5.

Absolute protein expression levels as MS signal intensities were estimated for proteins with at least two peptides in each bio-replicate of the respective gas mix by summing the five most intense product ions (of the most intense precursor) of the three most intense peptides (two if only two quantified). This combination has shown the highest accuracy for label-free absolute quantification from DIA MS data [67].

Differentially expressed proteins within SIL-protein-aided label-based quantification are presented in Additional file 4: Table S5, and within the proteome-wide label-free data set between syngas and CO or high-H_2_ CO and CO in Additional file 4: Tables S6, S7, respectively. Absolute protein expression levels are in Additional file 4: Table S8. The MS proteomics data have been deposited to the ProteomeXchange Consortium (http://proteomecentral.proteomexchange.org) via the PRIDE partner repository [68] with the data set identifier PXD008367.

### Genome-scale metabolic modelling

Model simulations were performed using GEM iCLAU786 of *C. autoethanogenum* [43] with modifications and simulation details specified in [28]. For simulations reported here, we used experimentally determined *C. autoethanogenum* biomass amino acid composition of high biomass syngas cultures reported in [28]. Biomass amino acid composition was determined at the Centre of Food and Fermentation Technologies (Tallinn, Estonia) using a method based on acid hydrolysis and LC–MS.

Briefly, we used FBA [44] to estimate intracellular fluxes (SIM1–19) and predict “optimal” growth phenotypes for our experimental conditions (SIM20–34) using either maximisation of ATP dissipation or biomass yield, respectively, as the objective function. In addition, for SIM35–41, CO_2_ reduction with the redox-consuming FdhA activity (reaction rxn00103_c0) was zeroed and the ratio between H_2_ utilisation for direct CO_2_ reduction (reaction rxn08518_c0), and Fd_red_ and NADPH generation (reaction leq000001) by the HytA–E/FdhA complex was fixed at a value corresponding to the respective experiment’s $q_{{{\text{H}}_{2} }}$/q_CO_ ratio (see [28] for details; both syngas and high-H_2_ CO data were used for fitting). Finally, we note that since carbon recoveries above 100% were observed, model input data for gas uptake rates were modified to achieve feasible solutions as specified in [28].

Simulation results identified as SIMx (e.g., SIM1) in the text are reported in Additional file 3: Tables S3, S4. The reactions together with their stoichiometries forming the metabolic network of GEM iCLAU786 can be found in Additional file 3: Table S4 and from the SBML model file of the GEM iCLAU786 in Additional file 5.

## Additional files

**Additional file 1.** Additional Figures S1–S7.

**Additional file 2.** Results of intracellular metabolome analysis. **Table S1.** Intracellular metabolite concentrations in high biomass cultures; **Table S2.** Metabolite abbreviations.

**Additional file 3.** Results of in silico analysis of experimental data and “optimal” growth predictions with the GEM iCLAU786. **Table S3.** Summary of results; **Table S4.** Complete results.

**Additional file 4.** Results of quantitative proteome analysis. **Table S5.** List of SIL-proteins with differential expression (*q* value < 0.05); **Tables S6, S7.** Proteins in proteome-wide data with differential expression (*q* value < 0.05 and a fold-change > 1.5) between syngas and CO, and high-H_2_ CO and CO, respectively; **Table S8.** Absolute protein expression levels as MS signal intensities.

**Additional file 5.** SBML file for the GEM iCLAU786.
