# Supplementary material for: H2 drives metabolic rearrangements in gas-fermenting Clostridium autoethanogenum
Source: Biotechnol Biofuels. 2018 Mar 1;11:55. doi: 10.1186/s13068-018-1052-9 (PMC5831606; doi:10.1186/s13068-018-1052-9)
Supplement: Supplementary file 1 — Additional file 1. Additional Figures S1–S7. [file 13068_2018_1052_MOESM1_ESM.pdf]

Additional file 1: Additional figures

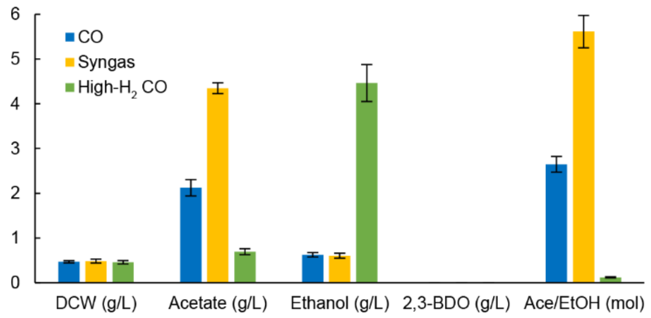

Fig. S1. Steady-state by-product concentrations in gas-fermenting *C. autoethanogenum* chemostats

Data for low biomass concentration chemostats (~0.5 gDCW/L) are shown and represented as the average ± standard deviation between biological quadruplicates. Syngas data from our previous work [28]. DCW, dry cell weight; Ace, acetate; EtOH, ethanol; 2,3-BDO, 2R,3R-butanediol.

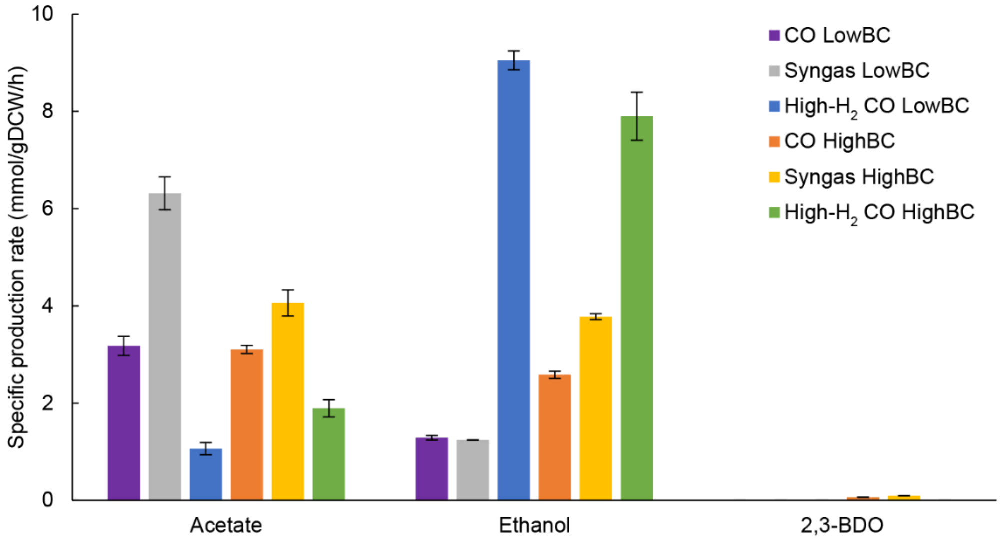

Fig. S2. Steady-state specific by-product production rates in gas-fermenting *C. autoethanogenum* chemostats

Data for both low (~0.5 gDCW/L) and high (~1.4 gDCW/L) biomass concentration (BC) chemostats are shown and represented as the average ± standard deviation between biological quadruplicates. Syngas data from our previous work [28]. DCW, dry cell weight; 2,3-BDO, 2R,3R-butanediol.

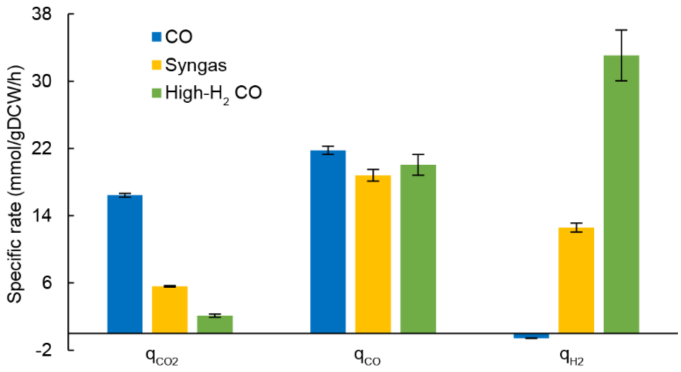

Fig. S3. Steady-state gas uptake and production in gas-fermenting *C. autoethanogenum* chemostats

Data for low biomass concentration chemostats (~0.5 gDCW/L) are shown and represented as the average ± standard deviation between biological duplicates (syngas), triplicates (high-H_2 CO), and quadruplicates (CO). Syngas data from our previous work [28]. DCW, dry cell weight; q_{CO2}, specific CO_2 production rate; q_{CO} and q_{H2}, specific CO and H_2 uptake rates, respectively.

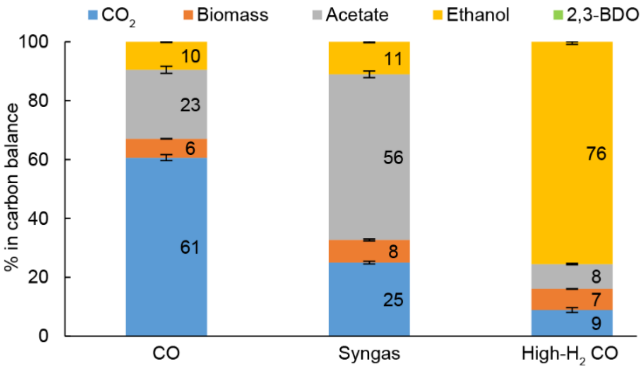

Fig. S4. Carbon balances of gas-fermenting *C. autoethanogenum* chemostats

Carbon recoveries of 121±5%, 115±1%, and 115±8% for CO, syngas, and high-H_2 CO, respectively, were normalised to 100% to have a fairer comparison of carbon distributions between the three gas mixes. Data for low biomass concentration chemostats (~0.5 gDCW/L) are shown and represented as the average ± standard deviation between biological duplicates (syngas), triplicates (high-H_2 CO), and quadruplicates (CO). Syngas data from our previous work [28]. 2,3-BDO, 2R,3R-butanediol.

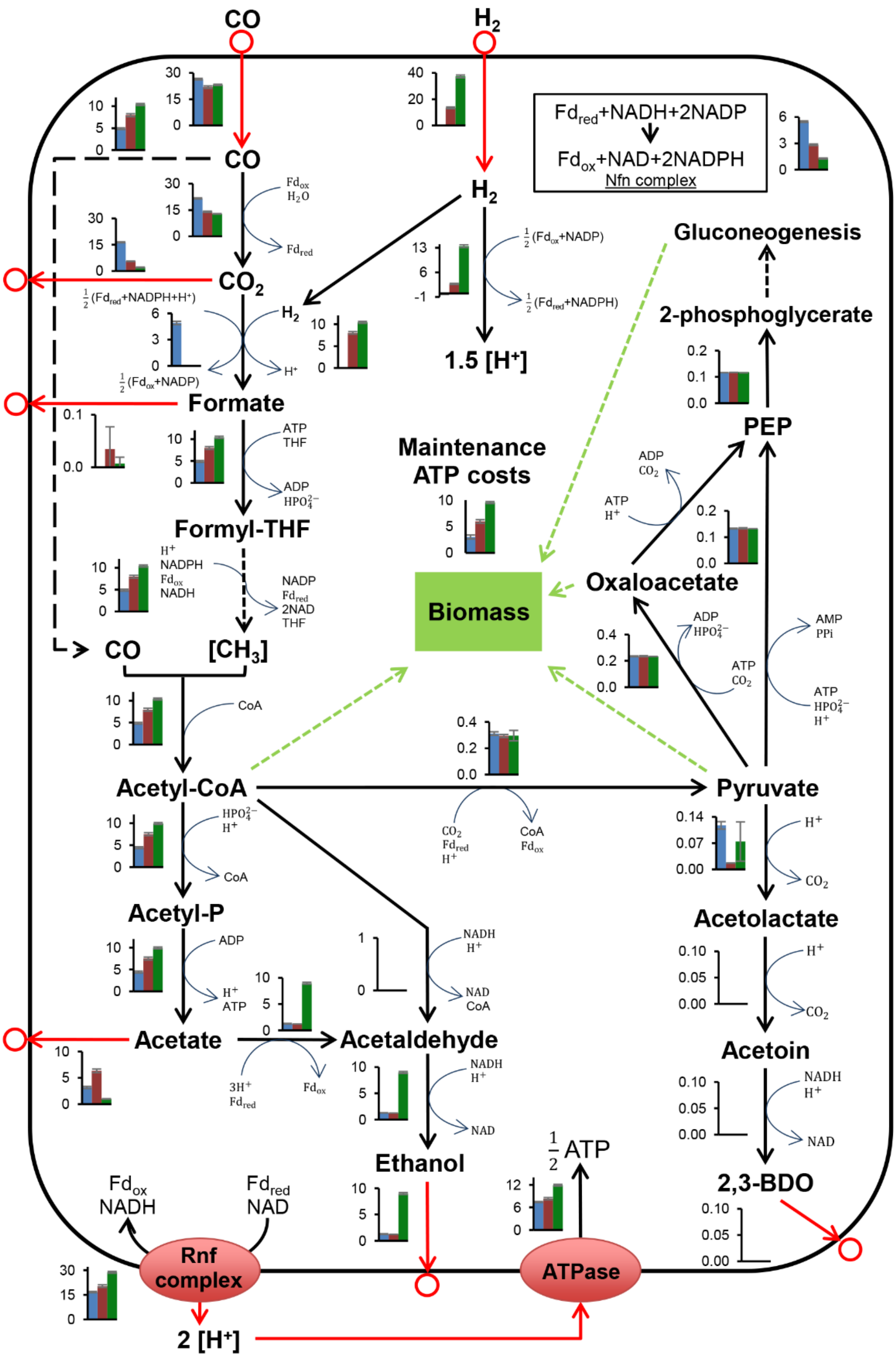

Fig. S5. Central metabolism flux levels of low biomass gas-fermenting *C. autoethanogenum* chemostats

Fluxes are represented as the average ± standard deviation for low biomass concentration (~0.5 gDCW/L) chemostats: blue bar, CO (quadruplicates); red, syngas (duplicate); green, high-H_2 CO (triplicate). Arrows show direction of calculated fluxes, red denotes uptake or secretion. Cofactors used in the GEM iCLAU786 are shown. Methylene-THF reductase flux is shown for the merged Formyl-THF-to-[CH3] step. Flux into PEP from oxaloacetate and pyruvate is merged. Flux unit is mmol/gDCW/h. gDCW, gram dry cell weight. Refer to Additional file 3: Tables S3 and S4 for data.

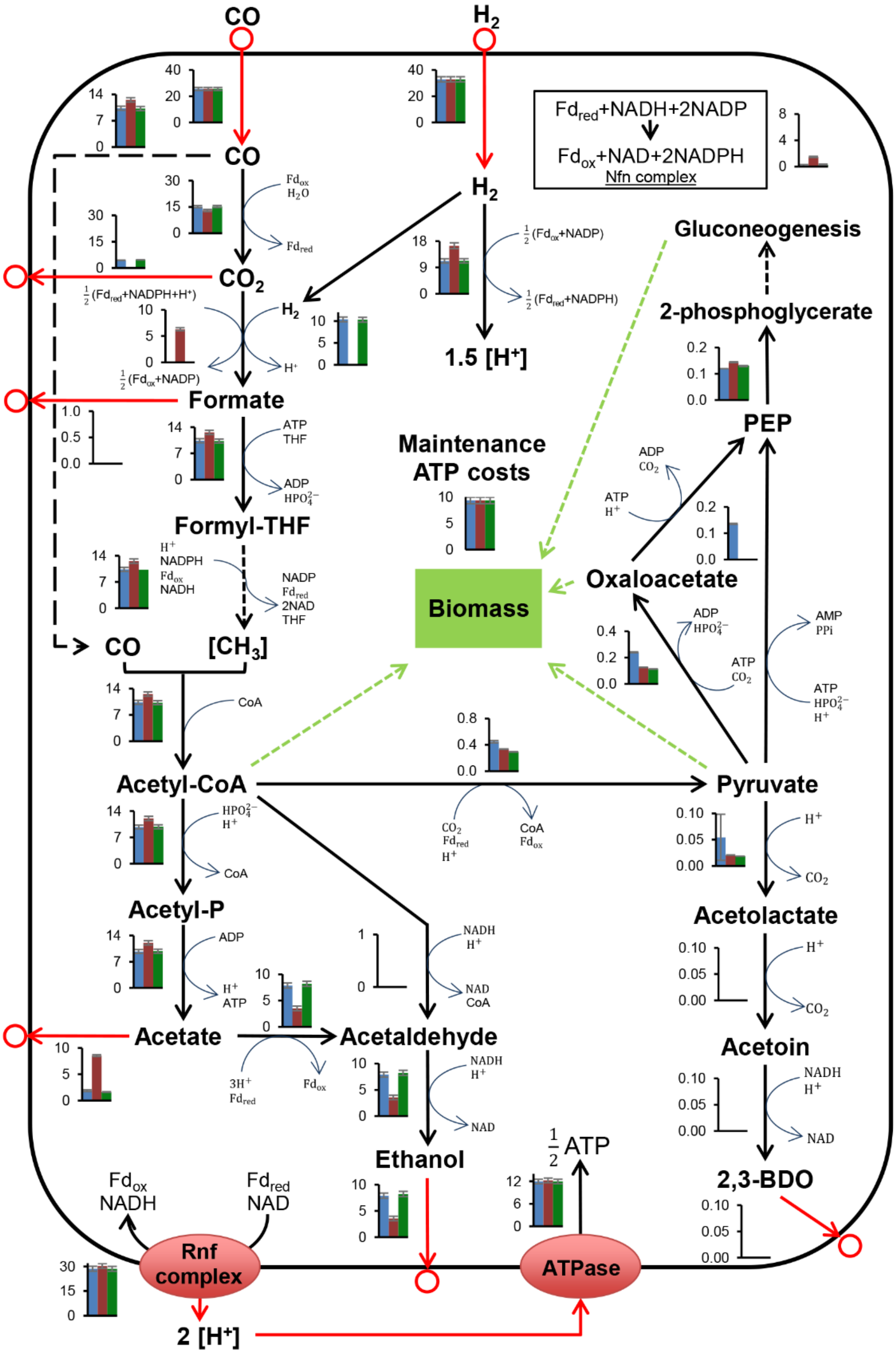

Fig. S6. Prediction of 'optimal' growth phenotypes for high biomass high-H_2 CO *C. autoethanogenum* chemostats using the GEM iCLAU786

Fluxes are represented as the average ± standard deviation between quadruplicate high biomass concentration (~1.4 gDCW/L) high-H_2 CO chemostats: blue bar, experimental fluxes; red, 'optimal' fluxes; green, 'optimal' fluxes with carbon and redox metabolism coupled from H_2 utilisation (see Text for details). Arrows show direction of calculated fluxes, red denotes uptake or secretion. Cofactors used in the GEM iCLAU786 are shown. Methylene-THF reductase flux is shown for the merged Formyl-THF-to-[CH3] step. Flux into PEP from oxaloacetate and pyruvate is merged. Flux unit is mmol/gDCW/h. gDCW, gram dry cell weight. Refer to Additional file 3: Tables S3 and S4 for data.

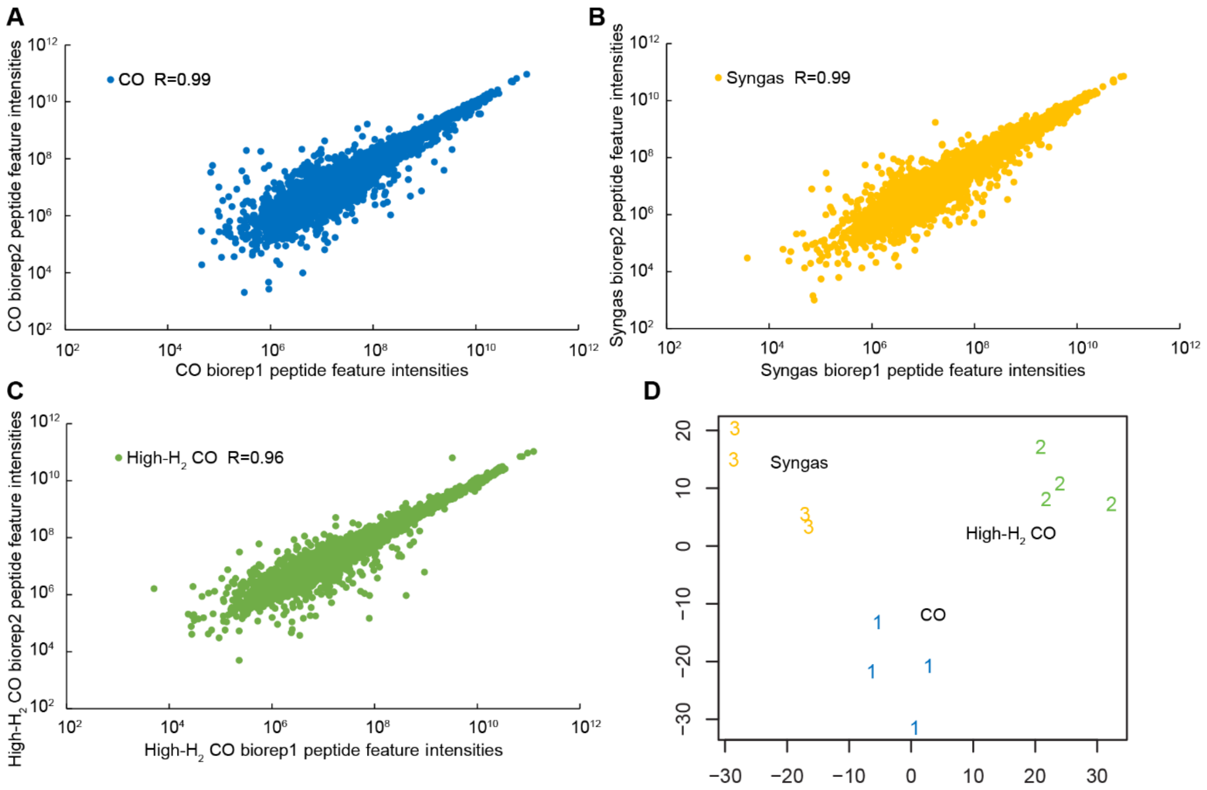

Fig. S7. Reproducibility and gas mix-specific clustering of proteomics data of gas-fermenting *C. autoethanogenum* chemostats

(A-C) Correlation between confidently quantified (q-value<0.01) peptide feature MS intensities of two exemplary bio-replicates. R, Pearson correlation coefficient. (D) Multidimensional scaling (MDS) plot of protein MS intensities of bio-replicates across all three gas mixes. Figure generated using the software Normalyzer [65].
